# Clinical technique for augmented reality‐guided tibial resection in kinematic alignment total knee arthroplasty: Real‐time ligament elongation enables personalised soft tissue balance

**DOI:** 10.1002/jeo2.70773

**Published:** 2026-06-16

**Authors:** Atsushi Sato, Masataka Ota, Toshiharu Miyazawa, Misako Takizawa, Reo Nagasaka, Marika Mukunoki, Kanako Izukashi, Jun Oike, Takayuki Okumo, Saki Yagura, Takayuki Koya, Koji Kanzaki

**Affiliations:** ^1^ Showa Medical University Fujigaoka Hospital, Orthopaedic Surgery Yokohama Japan; ^2^ Showa Medical University Koto Toyosu Hospital, Orthopaedic Surgery Tokyo Japan; ^3^ Showa Medical University School of Medicine, Physiology Tokyo Japan

**Keywords:** augmented reality navigation, kinematic alignment, ligament elongation pattern, soft‐tissue balance, total knee arthroplasty

## Abstract

**Purpose:**

Kinematic alignment (KA) total knee arthroplasty (TKA) aims to restore native knee kinematics by aligning prosthetic components with each patient's constitutional anatomy. While femoral resurfacing is well established, tibial resection in KA‐TKA remains largely subjective, particularly in cases with advanced deformity or bone loss. This study introduces a novel augmented reality (AR)–based technique for tibial resection that utilises real‐time ligament elongation patterns to support personalised alignment.

**Methods:**

More than 200 consecutive KA‐TKA procedures were performed using an AR navigation system (NextAR, Medacta) with medial‐stabilised implants (GMK Sphere). After femoral resurfacing and osteophyte removal, trial femoral components were inserted to restore functional intraoperative reference configuration, thereby defining a ‘zero‐position’ baseline. Real‐time elongation data of the medial and lateral collateral ligaments were visualised throughout the full range of motion via AR smart glasses. Tibial resection parameters—including varus/valgus angle and posterior slope—were quantitatively adjusted according to elongation patterns to replicate physiological soft‐tissue tension. Spacer blocks were used intraoperatively to verify bone‐gap balance.

**Results:**

Intraoperative tibial adjustments guided by ligament elongation patterns enabled patient‐specific resection strategies without reliance on ambiguous osseous landmarks or purely subjective gap assessments. This approach facilitated consistent intraoperative evaluation of medial and lateral soft‐tissue behaviour in both extension and flexion.

**Conclusion:**

This AR‐based KA‐TKA technique enables individualised tibial resection guided by real‐time ligament behaviour. It provides a reproducible and objective framework to support soft‐tissue‐informed intraoperative decision‐making, particularly in patients with complex deformities. This approach may complement existing KA principles and serve as a foundation for future outcome‐based investigations.

**Level of Evidence:**

NA.

**Abbreviations**
AR: augmented reality
CS: cruciate‐substituting
KA: kinematic alignment
LCL: lateral collateral ligament
MCL: medial collateral ligament
OR: operating room
TKA: total knee arthroplasty

## INTRODUCTION

Kinematic alignment (KA) total knee arthroplasty (TKA) is a patient‐specific surgical approach that aims to restore the native joint line orientation and soft‐tissue balance by aligning prosthetic components to each individual's constitutional anatomy, rather than to a neutral mechanical axis [3, 5, 6, 11, 15]. This strategy seeks to preserve natural knee kinematics and minimise the need for extensive soft‐tissue releases, which may be associated with improved functional outcomes and patient satisfaction [5, 6, 9].

Among the surgical techniques used to achieve KA, two principal methods have been described: the calipered technique and the manual‐in‐traction technique. The calipered technique, originally described by Howell et al., involves precise femoral resurfacing based on osseous landmarks, with bone resections matching implant thickness while accounting for cartilage wear and saw blade kerf [5, 6]. This technique aims to restore the three kinematic axes of the femur and avoids ligament releases. In contrast, the manual‐in‐traction technique determines the tibial resection through intraoperative gap assessment by applying axial traction and varus/valgus stress to balance extension and flexion spaces [3]. Although both techniques can achieve acceptable alignment, they remain largely subjective in determining tibial orientation in the coronal and sagittal planes.

In Asian populations, including Japanese patients, varus deformity due to osteoarthritis is often more pronounced than in Western populations [6, 14]. In addition, medial tibial bone loss is frequently encountered, reducing the reliability of osseous reference points in calipered techniques. Manual‐in‐traction methods, while functionally adaptive, are also influenced by intersurgeon variability in the direction and magnitude of traction force, leading to inconsistent gap assessment [3]. These challenges highlight the need for a reproducible, quantitative approach to determine tibial alignment, particularly in cases with advanced deformity or bone defects.

Biomechanical studies have shown that the medial collateral ligament (MCL) maintains near‐isometric length throughout the range of motion, whereas the lateral collateral ligament (LCL) shortens during flexion [2, 4, 8, 10]. These elongation patterns reflect physiological knee kinematics and may persist even after TKA when alignment approximates native anatomy [12]. Thus, intraoperative assessment of ligament elongation provides valuable insight into functional soft‐tissue balance [15].

To enable quantitative evaluation of ligament behaviour, we developed an intraoperative protocol that approximates a functional pre‐osteoarthritic condition by removing tibial osteophytes and inserting the femoral trial component after resurfacing. This configuration—defined as the zero position—should be interpreted as a functional intraoperative reference rather than a true restoration of pre‐arthritic anatomy. It serves as a reproducible biomechanical baseline from which real‐time ligament elongation can be quantified throughout flexion and extension. Using this reference state, surgeons can assess both medial and lateral ligament tension consistently, independent of deformity‐induced changes in bone geometry.

By quantifying these elongation patterns intraoperatively—particularly from the zero position—surgeons can tailor tibial alignment to support physiologic ligament balance. This approach enables personalised adjustment of tibial varus angle and posterior slope, supporting native medial stability and lateral rollback.

Recent advances in augmented reality (AR)–based navigation systems have made it possible to visualise such ligament elongation patterns directly in the operative field. These systems, such as the NextAR platform, have demonstrated high intraoperative bone resection accuracy, supporting their clinical reliability as precision tools for alignment optimisation [13].

Therefore, the present study describes a novel AR‐based tibial resection technique for KA‐TKA that allows real‐time, ligament‐based adjustment of tibial alignment using intraoperative ligament elongation patterns derived from a reproducible zero position. This manuscript is intended as a clinical technique paper, describing the surgical concept and intraoperative decision‐making framework enabled by AR, rather than an outcome‐based clinical study.

### System and implant

**Figure 1:**
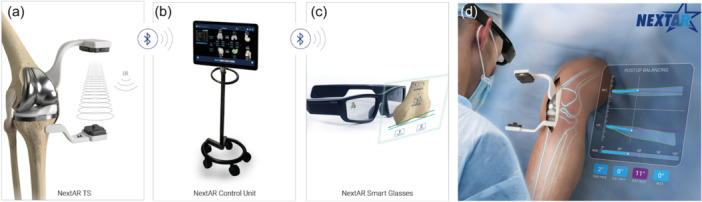
Augmented reality (AR) navigation system. System configuration of the AR navigation platform (NextAR, Medacta), showing key components including the tracking system (TS) (a), control unit (b), AR smart glasses (c) and intraoperative visualisation of alignment and ligament elongation patterns (d).

All procedures were performed using a computed tomography (CT)‐based AR navigation system, NextAR Knee (Medacta International). This system consists of a preoperative 3D planning platform, single‐use infrared sensors, a head‐mounted AR display (smart glasses) and a control unit. Real‐time intraoperative surgical information—including bone resection angles and soft tissue alignment—is visualised through the AR display, allowing for accurate, patient‐specific guidance of the surgical plan (Figure 1).

The prosthetic components used were from the GMK SPHERE total knee system (Medacta International), a medial‐pivot implant designed to restore physiological knee kinematics by providing enhanced medial stability and permitting unconstrained lateral motion.

### Technical background and surgical setting

The described surgical technique has been applied in more than 200 cases of primary TKA for varus knee osteoarthritis at our institution since the adoption of the NextAR system in 2022. Although no statistical analysis is presented in this report, the technique has been applied with consistent procedural feasibility and reproducibility. This technical note aims to describe the standardised procedural steps of this AR‐guided, ligament elongation‐informed tibial resection method in the context of kinematic alignment (KA) TKA.

### Preoperative planning

**Figure 2:**
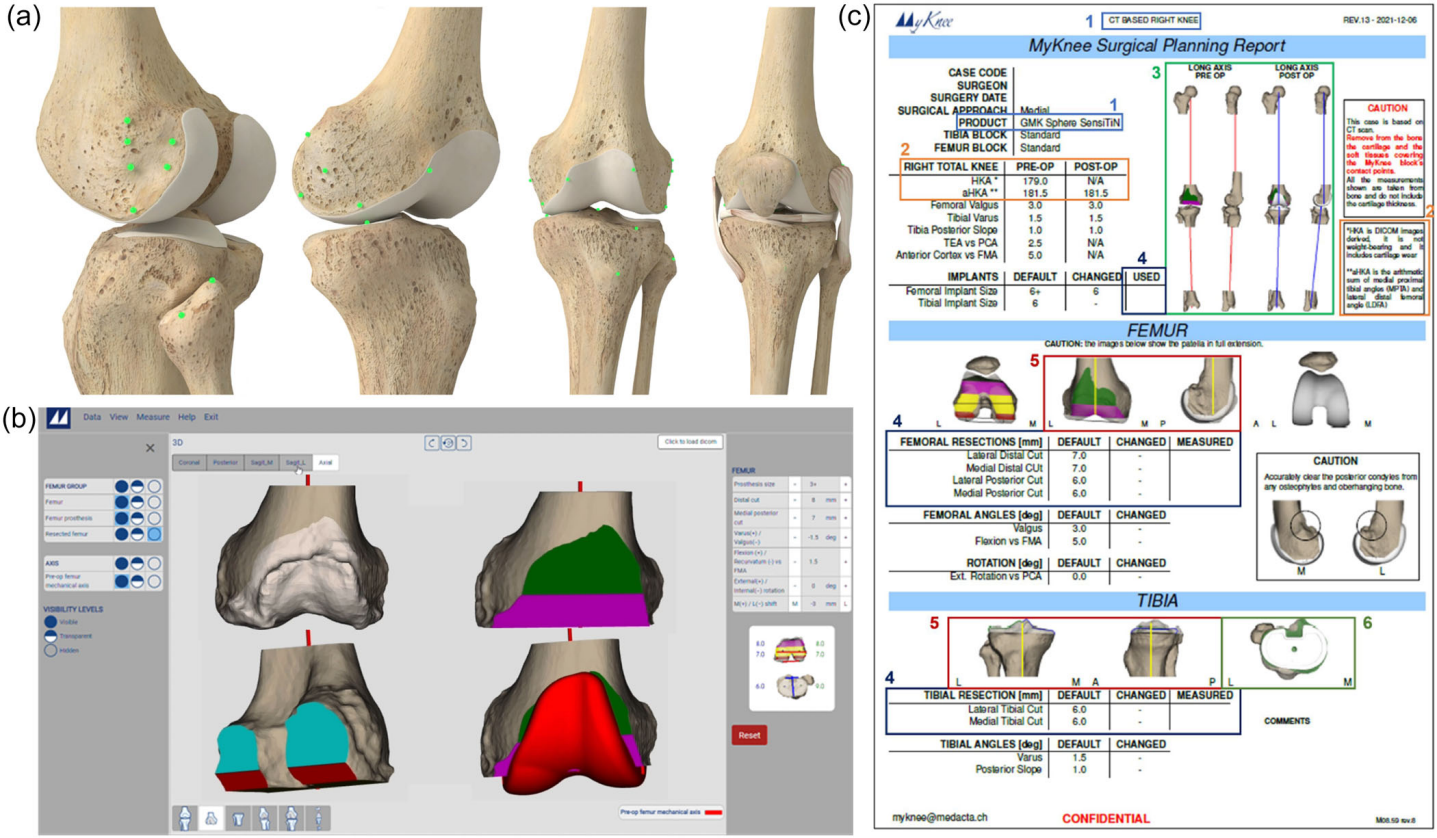
Preoperative planning. Preoperative planning using CT‐based 3D models and the MyKnee software (Medacta), including: (a) virtual 3D bone model and surface mapping; (b) resection planning for femur and tibia and (c) surgical planning report to guide kinematic alignment in total knee arthroplasty. CT, computed tomography.

All patients underwent full‐length CT imaging of the pelvis, knee and ankle for three‐dimensional preoperative planning. The NextAR software was used to evaluate the distal femoral and proximal tibial mechanical axes, joint line obliquity and posterior tibial slope. Additional alignment metrics, such as the coronal plane alignment of the knee classification and the arithmetic hip‐knee‐ankle angle, were assessed to guide the personalised surgical plan (Figure 2).

### Anaesthesia, surgical approach and AR system setup

All procedures were performed under general anaesthesia. An adductor canal block was routinely administered for perioperative analgesia. In some cases, a femoral nerve block was also performed at the discretion of the anesthesiologist. A standard medial parapatellar approach was used to access the knee joint. For AR‐based navigation, the MyKnee Pin Positioning System (Medacta International) was utilised to insert tracking pins for the NextAR system.

The femoral pin was placed intra‐articularly within the surgical field using the MyKnee system, whereas the tibial pin was typically inserted extra‐articularly outside the incision. In selected cases, the tibial pin was also placed intra‐articularly, depending on the patient's anatomy.

### Registration and initial intraoperative assessment

**Figure 3:**
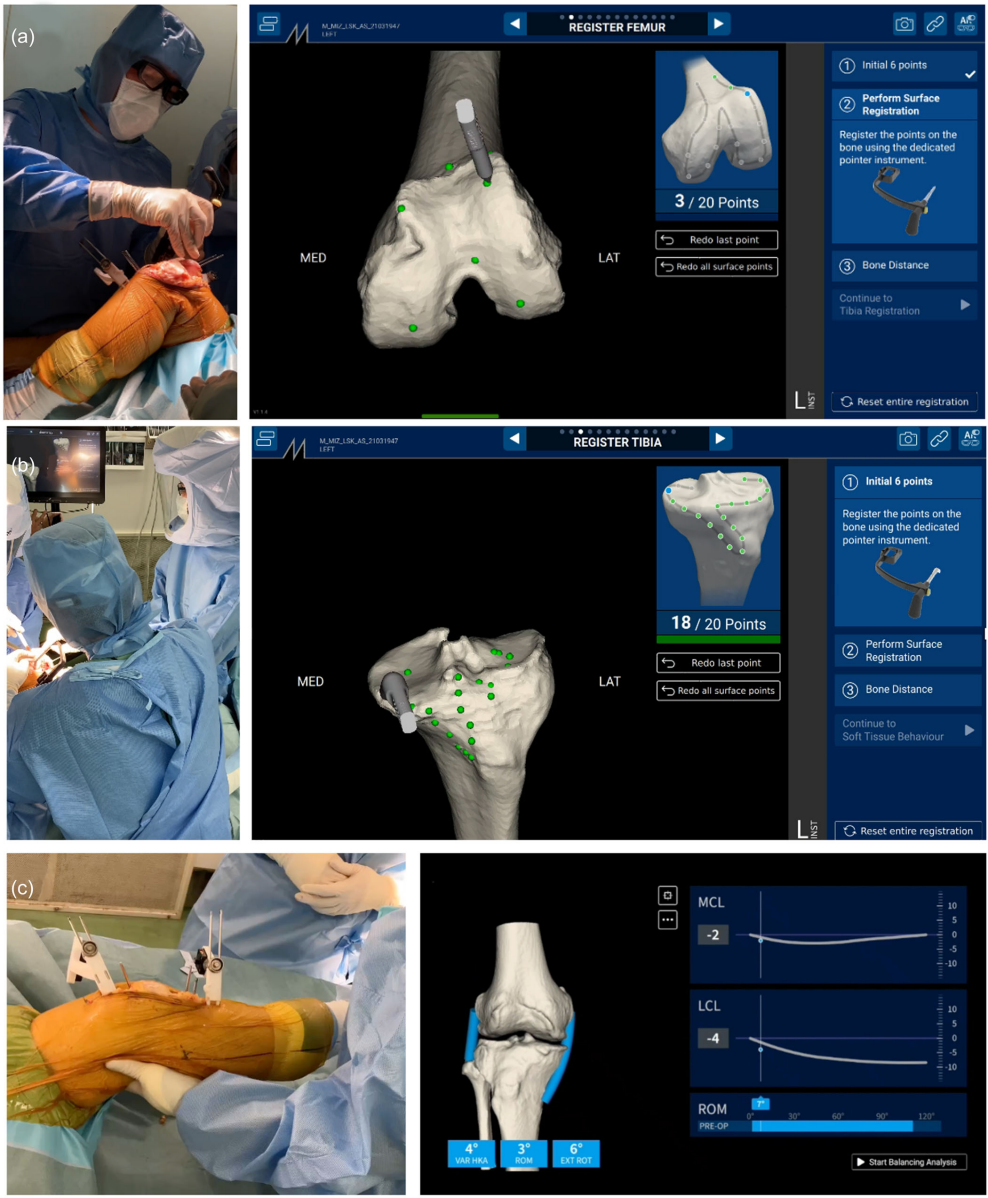
Registration and initial ligament elongation assessment. Intraoperative registration and initial zero‐position ligament elongation assessment under osteoarthritic joint conditions using the AR navigation system, including surface registration of the femur (a) and tibia (b) and (c) real‐time visualisation of medial (MCL) and lateral (LCL) ligament elongation patterns throughout the range of motion. AR, augmented reality; LCL, lateral collateral ligament; MCL, medial collateral ligament.

Following pin placement, a single‐use infrared camera and tracker were mounted onto the femoral and tibial MyKnee Pin Positioning Systems, respectively. The system was initialised to verify full‐range line‐of‐sight connectivity between the components, ensuring continuous mutual recognition throughout knee motion. The optimal positioning of the camera and tracker was confirmed via the external monitor prior to registration. Using a dedicated pointer, surface registration was performed to match the intraoperative anatomy with the preoperative CT data. The femur was registered by acquiring 6 anatomical landmark points, followed by 20 surface points. The tibia was registered using the same protocol. Once registration was complete, the system allowed real‐time measurement of ligament elongation patterns across the full range of motion, along with the definition of the medial and LCL boundaries (Figure 3).

In addition, the system enabled intraoperative calculation of tibial rotation relative to the femur, offering dynamic alignment feedback based on the actual ligament behaviour during knee flexion and extension.

### Femoral resection (calipered technique with AR guidance)

Both femoral and tibial resections were performed using an AR–guided adjustable cutting guide that allows intraoperative adjustment of varus–valgus and flexion–extension angles, as well as resection thickness in 1‐mm increments within a range of ±4 mm. Personalised patient‐specific cutting blocks were not used.

Femoral bone resections were performed according to the principles of the calipered technique in KA. The preoperative CT‐based plan provided by the NextAR system was used to determine the intended distal and posterior cutting angles. Using the AR smart glasses display, the cutting block was manually adjusted to match the planned resection angles in both the coronal and sagittal planes.

Once angular alignment was confirmed, an adjustable cutting block with ±4 mm attachments was utilised to achieve the planned bone resection depth. The resection thickness was matched to the prosthetic component's dimensions, incorporating estimated cartilage loss (typically 2 mm) and saw blade kerf (approximately 1 mm). Bone cuts were performed based on bone referencing, not soft tissue tension, to approximate the native joint line orientation based on bone referencing.

After resection, a dedicated verification device was used to confirm both the thickness and angular accuracy of the bone cut. If any discrepancy from the preoperative plan was noted, additional fine‐tuning cuts were performed to achieve the target resection.

### Femoral trial insertion and zero‐position setup

Following femoral resection, both the anterior and posterior cruciate ligaments (ACL and PCL) were excised, as this procedure was performed using a cruciate‐substituting (CS) implant design. Prominent tibial osteophytes were meticulously removed to eliminate mechanical distortion caused by osteoarthritic changes. This step was essential for approximating a functional pre‐osteoarthritic joint configuration and improving the accuracy of subsequent soft‐tissue assessments. The femoral trial component was then inserted to re‐establish the articular geometry of the distal femur. With the osteophytes removed and the femoral trial in place, the knee was taken through a full range of motion to evaluate patellar tracking, joint congruency and baseline ligament tension.

**Figure 4:**
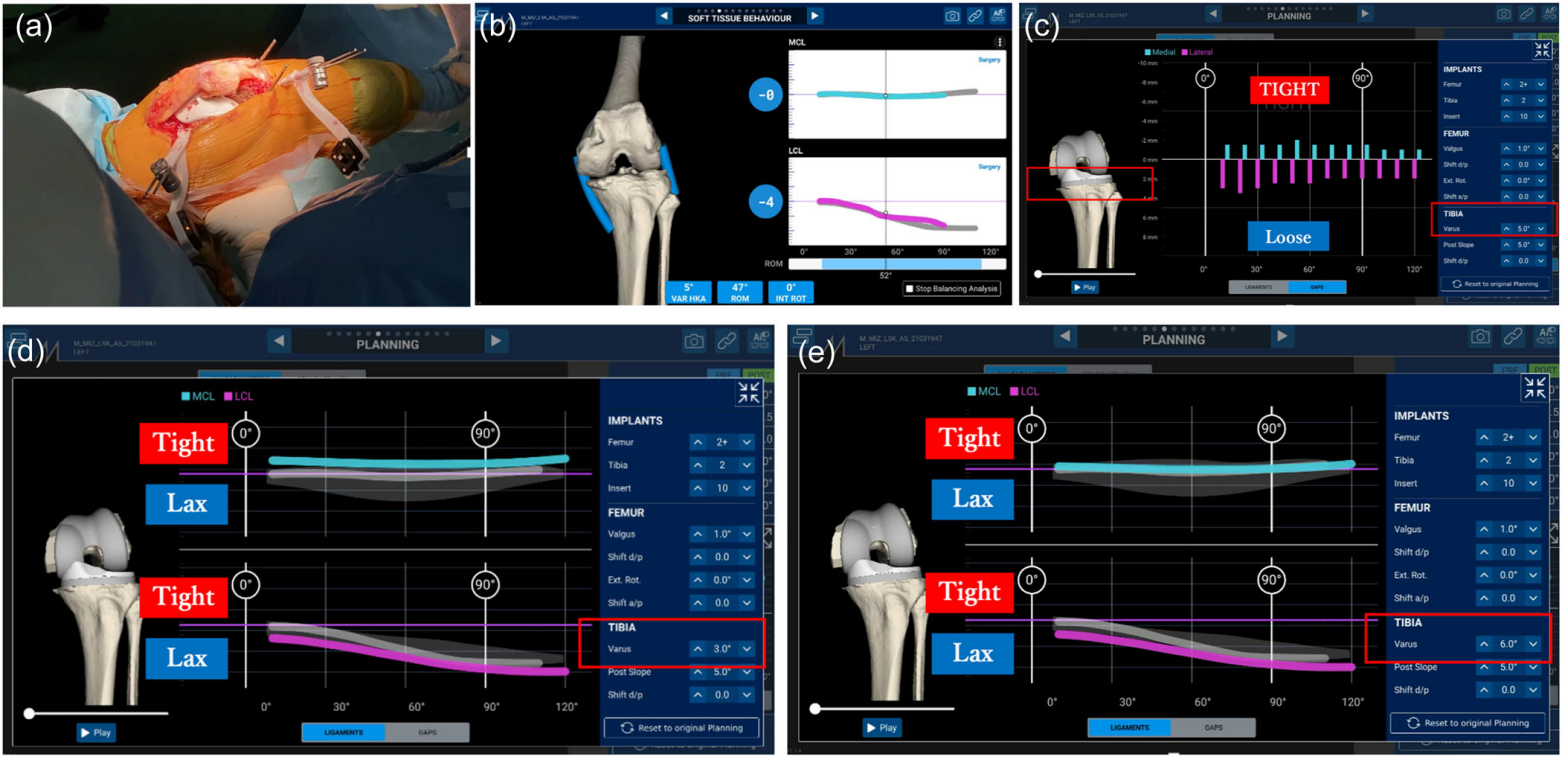
Femoral trial insertion and intraoperative tibial alignment adjustment. Femoral trial insertion and intraoperative adjustment of tibial alignment based on real‐time ligament elongation patterns. (a) Zero‐position measurement after femoral trial insertion and osteophyte removal (pre‐arthritic configuration). (b) Initial visualisation of MCL and LCL elongation curves. (c) Example of conventional gap assessment. Comparison of varus 3° (d) and varus 6° (e), demonstrating more physiological and consistent MCL elongation at 6°, which was selected for the final tibial resection. LCL, lateral collateral ligament; MCL, medial collateral ligament.

This intraoperative configuration—representing a functional intraoperative reference configuration—was defined as the ‘zero position’. It served as a reproducible biomechanical reference from which real‐time measurements of ligament elongation were obtained using the AR‐based navigation system. The medial and LCL lengths were automatically tracked throughout flexion and extension, providing dynamic and quantitative data on soft‐tissue behaviour. The zero position therefore enabled personalised adjustment of tibial alignment based on each patient's intrinsic ligament elongation pattern (Figure 4).

### Tibial resection based on ligament elongation pattern

Following femoral trial placement and osteophyte removal, a pre‐arthritic joint configuration—referred to as the ‘zero position’—was established as a functional intraoperative reference for soft‐tissue assessment. The AR‐based navigation system then enabled real‐time, quantitative assessment of medial and LCL elongation throughout the full range of motion, displayed directly through smart glasses. This setup allowed surgeons to continuously evaluate ligament behaviour without diverting their visual focus from the operative field.

Tibial resection parameters—including varus/valgus angle, posterior slope and resection depth—were adjusted according to the observed elongation patterns to approximate physiologic soft‐tissue tension (Figure 4). The specific intraoperative findings and corresponding adjustments were as follows:

- MCL/LCL tight in extension and flexion: Use a thinner liner or recut the tibia.
- MCL/LCL tight in flexion: Increase tibial posterior slope.
- MCL/LCL tight in extension: Remove posterior osteophytes and reassess MCL/LCL behaviour and joint gaps.
- MCL tight and LCL loose in extension: Recut the tibia in 1° more varus and select an insert 1 mm thicker as appropriate, then reassess MCL/LCL behaviour and joint gaps.
- MCL loose and LCL tight in extension: Recut the tibia in 1° more valgus and select an insert 1 mm thicker as appropriate, then reassess MCL/LCL behaviour and joint gaps.
- Complex imbalance in both compartments: Adjust alignment to achieve consistent medial tension in both extension and flexion.

These strategies are summarised in Table 1. In all cases, adjustments were guided not by fixed numerical targets but by dynamic feedback derived from each patient's native ligament behaviour. Although this soft‐tissue–driven approach was the primary determinant of tibial resection, bony gap balance was also confirmed intraoperatively using spacer blocks in both extension and flexion to ensure anatomical consistency with the observed elongation patterns. In addition, the system enabled real‐time assessment of joint gaps throughout the full range of motion, defined as the virtual distance between the distal‐most point of the femoral component and the lowest point of the tibial insert, which was referenced intraoperatively. Although these gap data were continuously monitored, previous studies have demonstrated that gap measurements do not necessarily correlate with ligament elongation, further emphasising the value of direct soft‐tissue assessment in KA‐TKA.

**Table 1:** Intraoperative findings and corresponding tibial resection adjustments based on ligament elongation patterns.

Intraoperative finding	Tibial adjustment	Rationale
MCL tight in extension and flexion	Increase tibial varus angle	Expands medial joint space and reduces medial soft tissue tension
MCL tight in flexion only	Increase posterior slope	Promotes posterior relaxation of medial structures during flexion
MCL loose in extension and flexion	Decrease tibial varus angle ± decrease resection level	Narrows medial joint space and increases medial tension
LCL tight in extension and flexion	Increase posterior slope ± additional tibial resection	Enhances lateral relaxation and reduces lateral tightness
LCL loose in extension and flexion	Decrease posterior slope ± decrease resection level	Tightens lateral soft tissue, especially when medial side is balanced
Complex imbalance in both compartments	Adjust alignment to achieve consistent medial tension in both extension and flexion	Medial stability is prioritised to ensure functional kinematics and prevent instability

*Note*: This table summarises the intraoperative rationale used to personalise tibial alignment based on real‐time ligament elongation patterns obtained via AR navigation in kinematic alignment TKA. Adjustments in tibial coronal alignment (varus/valgus), posterior slope and resection depth were tailored to specific soft tissue findings, rather than relying solely on bone gap measurements. In cases with complex imbalance, alignment was adjusted to maintain consistent medial tension throughout the range of motion, prioritising functional medial stability to restore native‐like kinematics and enhance postoperative joint stability. Abbreviations: LCL, lateral collateral ligament; MCL, medial collateral ligament; TKA, total knee arthroplasty.

### Final insert selection and implantation

After confirming satisfactory tibial resection and gap balance, the tibial trial baseplate, polyethylene insert trials of varying thicknesses and the femoral trial component were inserted. The AR‐based system was then used to re‐evaluate ligament elongation patterns and boundary behaviour throughout the full range of motion under trial conditions. Multiple insert thicknesses were tested to assess their effects on ligament behaviour and to determine the optimal insert that best approximated medial tension and lateral rollback patterns.

**Figure 5:**
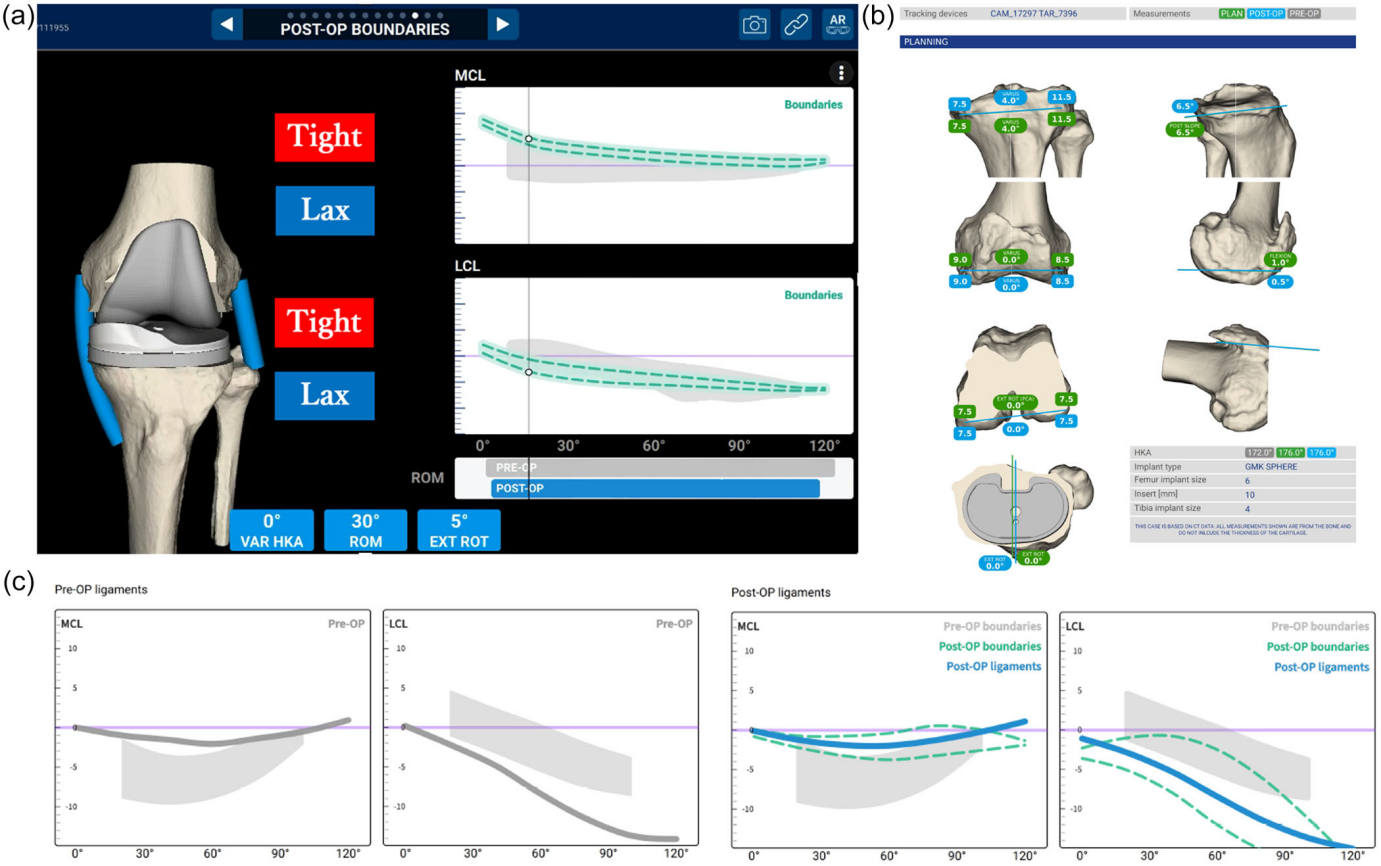
Postoperative ligament elongation and bone resection accuracy assessment. Postoperative visualisation of ligament elongation patterns and bone resection accuracy using the AR navigation system. (a) Medial (MCL) and lateral (LCL) elongation curves and boundaries after final implant insertion. (b) Verification of bone resection accuracy, with green indicating the preoperative plan and blue indicating the actual postoperative bone surface. (c) Comparison of preoperative and postoperative ligament elongation patterns for MCL and LCL. AR, augmented reality; LCL, lateral collateral ligament; MCL, medial collateral ligament.

Based on this assessment, the final insert thickness was selected to best reproduce physiologic soft‐tissue balance. The definitive femoral and tibial components were subsequently implanted using cemented fixation. Following implantation, the range of motion, joint stability and patellar tracking were reassessed to confirm appropriate kinematic function. The procedure was completed once final implant positioning and soft‐tissue balance were verified under functional conditions (Figure 5).

## DISCUSSION

This technical note introduces a novel tibial resection technique for KA TKA, utilising real‐time ligament elongation patterns visualised through an AR‐based navigation system. Unlike conventional calipered or manual‐in‐traction methods, this approach allows for quantitative, soft tissue‐driven adjustment of tibial alignment tailored to each patient's functional anatomy.

A key innovation of this technique is the intraoperative establishment of a ‘zero position’—a functional intraoperative reference configuration established after femoral resurfacing and osteophyte removal. This condition, minimally influenced by osteoarthritic deformity, provides a standardised reference for evaluating ligament elongation patterns throughout the range of motion. By anchoring tibial resection to this individualised reference, surgeons can interpret soft tissue tension in a physiologically meaningful way, supporting informed intraoperative decision‐making aimed at approximating native knee kinematics.

The importance of this approach is particularly evident in Asian populations, including Japanese patients, who often present with more severe varus deformities and proximal tibial bone defects compared to Western populations [7, 14]. These factors frequently compromise the reliability of osseous landmarks used in calipered techniques [5, 6]. Manual‐in‐traction methods, while more adaptable, remain susceptible to intersurgeon variability in traction direction and force, reducing reproducibility. By contrast, the elongation‐based method offers a reproducible, anatomy‐neutral solution that minimises subjectivity and enhances intraoperative precision.

The integration of AR smart glasses offers additional clinical benefits. Surgeons can assess ligament elongation and boundary behaviour directly within their operative field, eliminating the need to shift their gaze to external monitors. This hands‐free feedback enhances real‐time decision‐making and may shorten the learning curve for precise tibial alignment. The feasibility of AR–guided navigation in TKA has previously been reported by Fucentese and Koch, who demonstrated the potential of AR as a novel surgical guidance modality [1].

Although spacer blocks were used intraoperatively to confirm bone gap balance, the primary determinant of tibial resection in our technique was the real‐time ligament elongation pattern assessed through AR navigation. Previous studies have shown that collateral ligament elongation patterns reflect dynamic soft‐tissue behavior during knee motion, supporting the value of direct soft‐tissue assessment rather than relying solely on static geometric joint space evaluation [3, 15]. These findings underscore the clinical relevance of ligament elongation‐based assessment, which captures the dynamic behavior of soft tissues more directly than static gap measurements.

The accuracy of intraoperative bone resection using the AR–guided navigation system has already been quantitatively evaluated in a previous study, in which high agreement with the preoperative CT‐based plan was demonstrated [13]. While accurate bone resection is a prerequisite for precise component implantation, the present study intentionally focuses on the conceptual framework and intraoperative decision‐making process enabled by ligament elongation assessment, rather than duplicating validation data that have already been published.

Our technique prioritises functional soft tissue behaviour as the foundation for alignment decisions, which may better replicate physiological stability and potentially improve long‐term outcomes. It may be particularly beneficial in medial‐stabilised TKA designs such as GMK Sphere, where restoring appropriate medial tension and facilitating lateral rollback are crucial to reproducing native knee kinematics. Future studies may investigate the integration of this approach with sensor‐assisted devices or data‐driven algorithms to further personalise and optimise tibial alignment in kinematically aligned TKA.

## LIMITATIONS

This study has several limitations. First, it represents a technical report rather than a comparative clinical trial; therefore, no direct outcome data or statistical comparisons are presented. Although the technique has been applied in more than 200 cases with reproducible implementation, the functional and radiographic outcomes were not formally analysed.

Second, while ligament elongation patterns were used to guide tibial resection, the accuracy and validity of this approach depend on the correct identification of the ‘zero position’, which assumes restoration of the pre‐arthritic alignment following femoral resurfacing and osteophyte removal. However, because this procedure was performed using a CS implant design, both the ACL and PCL were excised. Therefore, the intraoperative condition cannot be regarded as a truly pre‐arthritic situation, which may limit the generalisability of this reference state. In cases of severe deformity or fixed contracture, establishing this reference may also be more challenging, particularly in knees that cannot achieve full extension, as this limitation could affect the accuracy of the zero position.

Finally, interpretation of elongation data and adjustment of tibial alignment angles still involve a certain degree of intraoperative decision‐making, although AR‐based guidance helps to standardise the workflow and minimise subjectivity. Accordingly, the zero position should be interpreted as a functional intraoperative reference rather than a true restoration of pre‐arthritic anatomy.

## CONCLUSION

We have presented a novel AR‐based tibial resection technique for kinematically aligned TKA, guided by real‐time intraoperative evaluation of ligament elongation patterns. By establishing a functional ‘zero position’ after femoral resurfacing, surgeons can perform patient‐specific adjustments to the tibial varus angle and posterior slope to restore physiologic soft‐tissue balance. This method addresses the limitations of conventional landmark‐ or gap‐based techniques, particularly in patients with severe deformity or bone defects. Incorporating ligament behaviour into alignment strategy may improve the personalisation and reproducibility of KA‐TKA, potentially contributing to better long‐term functional outcomes.

## AUTHOR CONTRIBUTIONS

Atsushi Sato conceptualised the study, developed the surgical technique and drafted the manuscript. Masataka Ota and Toshiharu Miyazawa contributed to intraoperative data acquisition and patient management. Misako Takizawa, Reo Nagasaka, Marika Mukunoki and Kanako Izukashi assisted with clinical coordination and postoperative data collection. Jun Oike and Takayuki Koya provided technical support in surgical navigation and contributed to data interpretation. Takayuki Okumo contributed to data interpretation and manuscript revision. Koji Kanzaki provided overall project supervision and approved the final manuscript. All authors read and approved the final version of the manuscript.

## FUNDING INFORMATION

The authors have no funding to report.

## CONFLICT OF INTEREST STATEMENT

The authors declare no conflicts of interest.

## ETHICS STATEMENT

This study was approved by the Institutional Review Board of Showa Medical University (IRB No. 2025‐0333; approval date: 17 October 2025). Written informed consent was obtained from all participants prior to inclusion in the study.

## Data Availability

The datasets generated and analysed during the current study are available from the corresponding author on reasonable request.
